# Experimental infections of rabbits with proliferative and latent stages of *Besnoitia besnoiti*

**DOI:** 10.1007/s00436-015-4612-y

**Published:** 2015-07-07

**Authors:** Emmanuel Liénard, Loredana Pop, Françoise Prevot, Christelle Grisez, Virginie Mallet, Isabelle Raymond-Letron, Émilie Bouhsira, Michel Franc, Philippe Jacquiet

**Affiliations:** UMR INRA/DGER 1225, INP - École Nationale Vétérinaire de Toulouse, Laboratoire de Parasitologie, 23 chemin des Capelles, F-31076 Toulouse, France; Université de Toulouse, Institut National Polytechnique de Toulouse, École Nationale Vétérinaire de Toulouse, Laboratoire de Parasitologie et Maladies Parasitaires, 23 chemin des Capelles, F-31076 Toulouse, France; Parasitology and Parasitic Diseases Department, Faculty of Veterinary Medicine, University of Agricultural Science and Veterinary Medicine Cluj-Napoca, 3-5 Calea Mănăştur, 400372 Cluj-Napoca, Romania; Université de Toulouse, Institut National Polytechnique, École Nationale Vétérinaire de Toulouse, Laboratoire d’histopathologie expérimentale et comparée, 23 chemin des Capelles, F-31076 Toulouse, France; Stroma Lab, UMR 5273 UPS EFS INSERM 1031, 1 avenue Jean Poulhes, F-31403 Toulouse, France

**Keywords:** Cattle besnoitiosis, Rabbit model, *Besnoitia besnoiti* cysts, qPCR, IFAT, Immunoblot

## Abstract

Cattle besnoitiosis due to *Besnoitia besnoiti* is spreading across Europe and is responsible for severe economic losses in newly infected herds. Experimentally speaking, rabbits have been found to be susceptible to this parasite. The adaptation of *B. besnoiti* to rabbits may offer a new, easier and cheaper model of investigation for this disease. This study compared the virulence between tachyzoites and bradyzoites of *B. besnoiti* in rabbits. Eighteen New Zealand rabbits were allocated into three groups of six animals each. The rabbits from the control (group C), “tachyzoite” (group T) and “bradyzoite” (group B) groups were subcutaneously injected in the right flank with 66 μg of ovalbumin, 6.10^6^ tachyzoites (125th passage on Vero cells) and 6.10^6^ bradyzoites (collected from a natural infected cow) of *B. besnoiti*, respectively. Clinical follow-up and blood sampling for serological survey and qPCR were performed during 10 weeks until euthanasia. Molecular and immunohistochemistry examination was achieved on 25 samples of tissue per rabbit. Seroconversion occurred in group T without any clinical signs. Rabbits of group B exhibited a febrile condition (temperature above 40 °C from day 8 to day 11 following injection) with positive qPCR in blood. Cysts of *B. besnoiti* were found on skin samples and organs of rabbits from group B in tissue explored with threshold cycle (Ct) values below 30. These results suggest a higher virulence of bradyzoites in rabbits than Vero cell-cultivated tachyzoites. The proposed model could be used to assess the in vivo effectiveness of vaccine or drugs against cattle besnoitiosis.

## Introduction

Cattle besnoitiosis is caused by a protozoan parasite *Besnoitia besnoiti*, a cyst-forming Apicomplexa intimately related to *Toxoplasma gondii* and *Neospora caninum* (Ellis et al. 2000). This disease is widespread in Africa, Asia and the southwest of Europe (Jacquiet et al. 2010). Considered as an emerging disease in cattle according to the European Food Safety Authority (EFSA 2010), recent outbreaks have been reported in Germany (Mehlhorn et al. 2009), Italy (Mutinelli et al. 2011; Gentile et al. 2012), Switzerland (Lesser et al. 2012), Croatia (Cortes et al. 2014) and in Hungary (Hornok et al. 2014). The classical course of the disease in cattle is divided into three successive clinical phases (Jacquiet et al. 2010). The initial febrile stage is non-specific with ocular and nasal discharges. The rapid multiplication of tachyzoites occurs within bovine macrophages and endothelial cells of the blood vessels. It is followed by generalized edema. Then, the final chronic phase arises with scleroderma and development of many thousands of pinhead-sized, thick-walled cysts containing bradyzoites in various tissues including the skin. Severe economic losses, especially in newly affected farms, are mainly due to fertility issues in bulls, weight loss, mortality and leather and carcass depreciations (Pols 1960; Cortes et al. 2006b; Álvarez-García et al. 2013). Only a few infected animals exhibit clinical signs in affected herds (Bigalke 1968; Jacquiet et al. 2010). The epidemiological role of seropositive but subclinically infected cattle as a potential source of parasite should be further investigated (Bigalke 1968; Frey et al. 2013; Liénard et al. 2013b). Numerous features of this disease remain currently enigmatic. The life cycle of *B. besnoiti* is not yet described, and the final host was not identified (Diesing et al. 1988; Olias et al. 2011; Basso et al. 2011; Álvarez-García et al. 2013). Causes of its current expansion are not clearly explained. Intense animal trade throughout countries could be the major contribution to parasite dissemination and founding of new cattle besnoitiosis foci (Olias et al. 2011). Some evidence has been collected about its mechanical and horizontal vector-borne transmission ability through hematophagous arthropods (Bigalke 1968; Liénard et al. 2013a). This route could act in local transmission within herd or between close herds as well as repeated use of infected hypodermic needles (Jacquiet et al. 2010; Álvarez-García et al. 2013). Moreover, the absence of *B. besnoiti* DNA in the semen from infected bulls makes the sexual transmission of bovine besnoitiosis unlikely under natural conditions (Esteban-Gil et al. 2014).

Several laboratory rodents (hamsters, gerbils, guinea pigs, common voles, rats, various strains of white mice: NMRI mice, GKO mice) have been experimentally infected with *B. besnoiti* (Pols 1960; Neuman and Nobel 1981; Shkap et al. 1987; Schares et al. 2009; Basso et al. 2011). The clinical course is variable, from no clinical to rapid death, but none of them have developed cysts in their tissue. Experimentally, rabbits have been shown to be susceptible to *B. besnoiti* (Pols 1954a, b; Bigalke 1968; Basson et al. 1970). The acute phase was evident, but the chronic stage was not as severe as for cattle. No scleroderma has been reported in rabbits, and the maturation of *B. besnoiti* cysts in skin is erratic (Pols 1954a, b; Bigalke 1968; Basson et al. 1970; Cortes et al. 2006b; Basso et al. 2011). When they were present, the number of cysts recovered in skin samples was lower than in cattle and no scleroderma was noticed (Pols 1954a, b; Bigalke 1968; Basson et al. 1970). Since those investigations, no cysts were found in rabbits after experimental inoculations of bradyzoites or tachyzoites of *B. besnoiti* using various doses and routes (Shkap et al. 1987; Cortes et al. 2006b; Basso et al. 2011). However, the adaptation of *B. besnoiti* to rabbits may offer a new, easier and cheaper model of investigation for cattle besnoitiosis regarding, for example, host immune response or in vivo therapeutic assays (Moine et al. 2015) and vaccine trials against this disease. Indeed, the treatment is at the moment not satisfactory in cattle. It is based on high-level dose of sulfamides for at least 7 days. It is partially effective during the acute stage and fails to cure infected cattle completely (Jacquiet et al. 2010). Moreover, no vaccine is licensed in Europe.

Thus, facing these discrepancies, the first aim of the present study was to determine the susceptibility of rabbits subcutaneously inoculated with either Vero cell-cultivated tachyzoites or bradyzoites collected from a naturally infected cow using clinical, serological, histopathological and molecular tools. Moreover, the virulence can be attenuated in some apicomplexan parasites such as *T. gondii* or *N. caninum*. Continuous passage on mice of the S48 strain of *T. gondii* over 3000 times has led to the development of a commercial live vaccine labelled Toxovax® in sheep (Buxton 1993). The pathogenicity of *N. caninum* dramatically decreased following in vitro culture of murine macrophage (J774) cell lines (Khordadmehr et al. 2013). Then, the second aim of this study was to evaluate the difference of pathogenicity between bradyzoites and long-term Vero cell-cultured parasites in rabbits.

## Material and methods

### Animals

Eighteen 9-month-old male (8) and female (10) New Zealand White rabbits were purchased from the French National Institute of Agricultural Research (Montgiscard, France). They weighed between 3.5 and 4.5 kg at the start of the study. The animals were handled in strict accordance with good animal practice as defined by the relevant European standards of welfare for animals in research in authorized facilities (agreement no. C31 555 11). The rabbits were acclimatized for 3 weeks prior to being assigned randomly to three groups of six animals that were housed in individual boxes. They were fed exclusively commercial food and tap water ad libitum.

### Source of *Besnoitia besnoiti* bradyzoites and preparation of inoculations

The source of bradyzoites was a 4-year-old cow with chronic besnoitiosis coming from a commercial herd (south-west of France). Its serological status was positive after evaluation by Western blot as previously described by Liénard et al. (2013a). The cow was sedated by intramuscular injection of xylazine (Rompun®, Bayer Santé Division animale, Puteaux, France) at a dose rate of 0.3 mg/kg. A skin sample from the back line was removed for the isolation of *B. besnoiti* bradyzoites. This skin location exhibited threshold cycle (Ct) values of about 16 corresponding to a significant parasite burden. The cow was then immediately euthanized by intravenous injection of 0.12 ml/kg BW of T-61® (T-61®, MSD Santé animale, Beaucouzé, France). Bradyzoites of *B. besnoiti* were freed from tissue cysts with a sterile scalpel in a Petri dish containing phosphate-buffered saline (PBS) (Bio-Rad, Marnes-la-Coquette, France) plus 500 U penicillin/ml, 500 μg streptomycin/ml (GIBCO Pen Strep®, Life Technologies™, Saint-Aubin, France) and 500 U nystatin/ml (Sigma-Aldrich, Saint-Quentin Fallavier, France). This mix was filtered through a 40-μm cell stainer (BD Falcon® 40 μm, BD Biosciences Discovery Labware, Rembodegen, Belgium) and centrifuged at 2000 rpm for 10 min at 4 °C. The pellet was resuspended in 1 ml of PBS, and bradyzoites were counted with a hemocytometer (Merck, Fontenay-sous-Bois, France). The concentration of the parasite was adjusted with PBS to prepare six doses with a final volume of 500 μl containing 6.10^6^ bradyzoites.

### Source of *Besnoitia besnoiti* tachyzoites

Purified *B. besnoiti* tachyzoites from a strain isolated in the French Pyrenees (Liénard et al. 2013a) were used for preparation of infective doses to rabbits and as source of antigens for the Western blot (WB) and the immunofluorescence antibody test (IFAT). This isolate of *B. besnoiti* has been cultivated on Vero cells at the E.N.V.T. since 2011. The tachyzoites were at the weekly passage 125. Free tachyzoites in suspension were collected in PBS and centrifuged at 2000 rpm for 10 min at 4 °C. Pellets of tachyzoites were resuspended in PBS. The concentration was adjusted to prepare six doses of 6.10^6^ tachyzoites in 500 μl of PBS.

### Experimental design

The control group (group C) received a subcutaneous injection of 66 μg of ovalbumin at the right flank (Sigma-Aldrich, Saint-Quentin Fallavier, France) equivalent to the amount of proteins of 6.10^6^*B. besnoiti*. The rabbits from the “tachyzoites” (group T) and “bradyzoites” (group B) groups were subcutaneously injected in the right flank with individual dose of 6.10^6^ tachyzoites or bradyzoites, respectively.

### Clinical examination

All animals were clinically monitored at day 4 before the parasite inoculation and every day from day 0 to day 21 and then every 2 or 3 days until day 70. Rectal temperatures were recorded according to the same regimen. Rabbits were euthanized at day 70 post-inoculation by injection into the marginal ear vein of 0.12 ml/kg BW of T-61®.

### Blood samples, timing of serological and qPCR examinations on blood

Blood was taken from the cephalic vein on days –4, 2, 7, 14, 21, 28, 35, 42, 49, 56, 63 and 70. The blood was collected in 3-ml tubes containing EDTA (Terumo Europe N.V., Leuven, Belgium) for assessment of parasite load by qPCR. Serum collected in 3.5-ml tubes containing silicone (Terumo Europe N.V., Leuven, Belgium) was tested for *B. besnoiti* antibodies by WB using *B. besnoiti* tachyzoite antigens and by the indirect fluorescent antibody test (IFAT). Additional blood samplings were performed on days 9 and 17 on group B for qPCR examination.

### Western blot procedures

WB procedures, adapted from Cortes et al. (2006a), were performed as detailed by Liénard et al. (2011). Sera were tested at a 1:50 dilution. Peroxidase-labelled goat anti-rabbit IgG conjugate (Anti-Rabbit IgG (Whole molecule)-Peroxidase, Sigma-Aldrich, Saint-Quentin Fallavier, France) was used at 1:150 dilution. Serum from one rabbit infected with bradyzoites was used as positive control. A serum was considered positive when at least four of ten bands of specific tachyzoite antigens (45, 40, 37, 34, 30, 27, 22, 17, 16, and 15 kDa) were observed (Schares et al. 2010). Negative control was obtained from ovalbumin-inoculated control rabbits.

### Indirect fluorescent antibody test

The procedures are described by Lenfant et al. (2014) and were adapted to rabbit sera. Serum samples were tested at serial twofold dilutions from 1:100 to 1:6400. Fluorescein isothiocyanate (FITC)-labelled goat anti-rabbit IgG conjugate (Anti-Rabbit IgG (Whole molecule)-FITC Antibody (goat), Sigma-Aldrich, Saint-Quentin Fallavier, France) was used at a 1:200 dilution in PBS with 0.05 % Evans blue. The slides were read under a transmitted light fluorescence microscope (AxioScopeA1, Carl Zeiss SAS, Marly le Roi, France) at ×400 magnification by one observer. Unbroken, peripheral bright fluorescence of the tachyzoite membrane at a 1:200 dilution was considered the positive cut-off (Shkap et al. 2002; Lenfant et al. 2014).

### Necropsy and qPCR on blood and tissues

At D70, necropsies of all animals were performed. For each animal, 25 skin and tissue samples were collected for PCR and histological analyses. Skin samples were taken from the right fore and hind limbs, the scrotum (males), right inner thigh, backline, right flank (i.e. inoculation site), umbilicus area, udder, right neck, right shoulder and right eyelid. For tissues and organs, samples were taken from the right abdominal platysma, pancreas, liver, spleen, right kidney, gallbladder, diaphragm, heart, lung, nasal and tracheal mucosa, right eye, right testicle or right ovary, penis or vulvar mucosa and vaginal mucosa.

Quantitative PCR was used to detect *B. besnoiti* DNA from skin, tissue and blood samples of the rabbits. DNA (50 mg samples or 1 ml of blood) was extracted with the QIAmp® DNA Mini Kit (Qiagen, Courtaboeuf, France) commercial kit, according to the manufacturer’s recommendations. *B. besnoiti* ITS-1 amplification was performed with the commercial PCR kit AdiaVet™ Besnoitia (AES Chemunex, Bruz, France). The quantitative PCR was performed with the Stratagene MX3005P thermal cycler (Agilent Technologies, La Jolla, CA), and results were analysed using the MxPro QPCR version 4.10 software (Agilent Technologies, La Jolla, CA). A Ct value of 40 corresponded to a negative result.

### Immunohistopathological examination

To evaluate the presence of *B. besnoiti* cysts, mirror samples of qPCR-positive locations with Ct values below 30 were fixed in 10 % phosphate-buffered formalin. They were dehydrated and embedded in paraffin wax at 56 °C, sectioned at 4 μm and stained with haematoxylin and eosin for conventional evaluation. Immunohistochemistry was performed by a peroxidase-based staining method, using polyclonal antibodies obtained from a naturally infected cow by *B. besnoiti* and further characterized by WB and IFAT (Liénard et al. 2013a). After 1-h incubation with these polyclonal antibodies (dilution 1/25), tissue slides were incubated 25 min with antibovine peroxydase solution at a dilution rate of 1/1000 (antibovine IgG (whole molecule)−peroxidase antibody produced in rabbit, Sigma-Aldrich, Saint-Quentin Fallavier, France). Peroxydase activity was revealed with diaminobenzidine as chromogen (DAB+, Dako France SAS., Les Ulis Cedex, France), and slides were counterstained with Harris haematoxylin.

### Statistical analyses

For the rectal temperature, pairwise comparisons between experimental groups were performed using the exact procedure of permutation test with the Bonferroni correction. The Wilcoxon-Mann-Whitney test with the exact procedure was used to compare antibody titres determined by IFAT between groups B and T with the Bonferroni correction. The mean Ct values found in the tissues and skin were also compared using permutation test with the exact procedure. All statistical analyses were carried out with the software package StatXact® release -10 (Cytel Software Corporation, USA). For all analyses, values of *p* < 0.05 were considered significant.

## Results

### Clinical follow-up

**Fig. 1:**
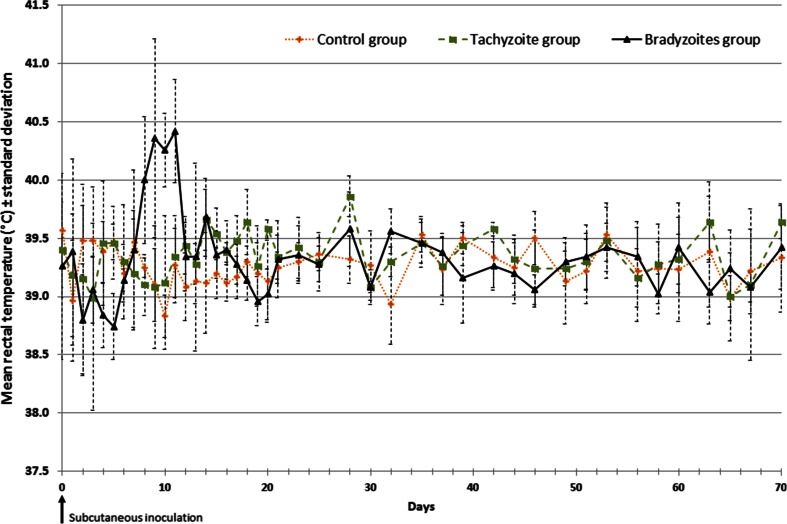
Evolution of mean rectal temperatures (± *standard deviation bars*) in each rabbit group. The control, tachyzoite and bradyzoite groups were subcutaneously challenged with 66 μg of ovalbumin, 6.10^6^ tachyzoites and 6.10^6^ bradyzoites of *B. besnoiti* at day 0, respectively

No clinical abnormality was observed in rabbits belonging to group C during the whole experimental period and following the injection of ovalbumin. No increase of rectal temperature was found (Fig. 1). A male rabbit (T05) from group T died 3 days after the inoculation of tachyzoites without any clinical sign and fever. The necropsy revealed sudden pancreatitis. For the five remaining rabbits, none of them showed clinical signs, or significant changes in rectal temperature (Fig. 1), at any time-point during the experiment. One male rabbit (B03) died 1 day after the injection of bradyzoites without any clinical signs. No clinical abnormality was found after the necropsy of this rabbit. The five remaining rabbits of group B exhibited fever which started 7 days post-infection (dpi) and lasting 4 days before back to initial values at day 12 (Fig. 1). Rectal temperatures increased to 40.4 °C (ranging from 39.1 to 41.1 °C at 9 and 11 dpi) after returning to normal values (Fig. 1). From days 8 to 10, the rise of rectal temperature was significant in comparison to group C (day 8: *p* = 0.0303, day 9: *p* = 0.0173, day 10: *p* = 0.0043, day 11: *p* = 0.0043) and group T (day 8: *p* = 0.0238, day 9: *p* = 0.0317, day 10: *p* = 0.00794, day 11: *p* = 0.00794). In the same period, no significant differences of temperatures were observed between group C and group T. It was accompanied by photophobia for all rabbits in group B from day 8 to day 10. One rabbit exhibited a bilateral nasal discharge at day 7.

### Antibody response

**Fig. 2:**
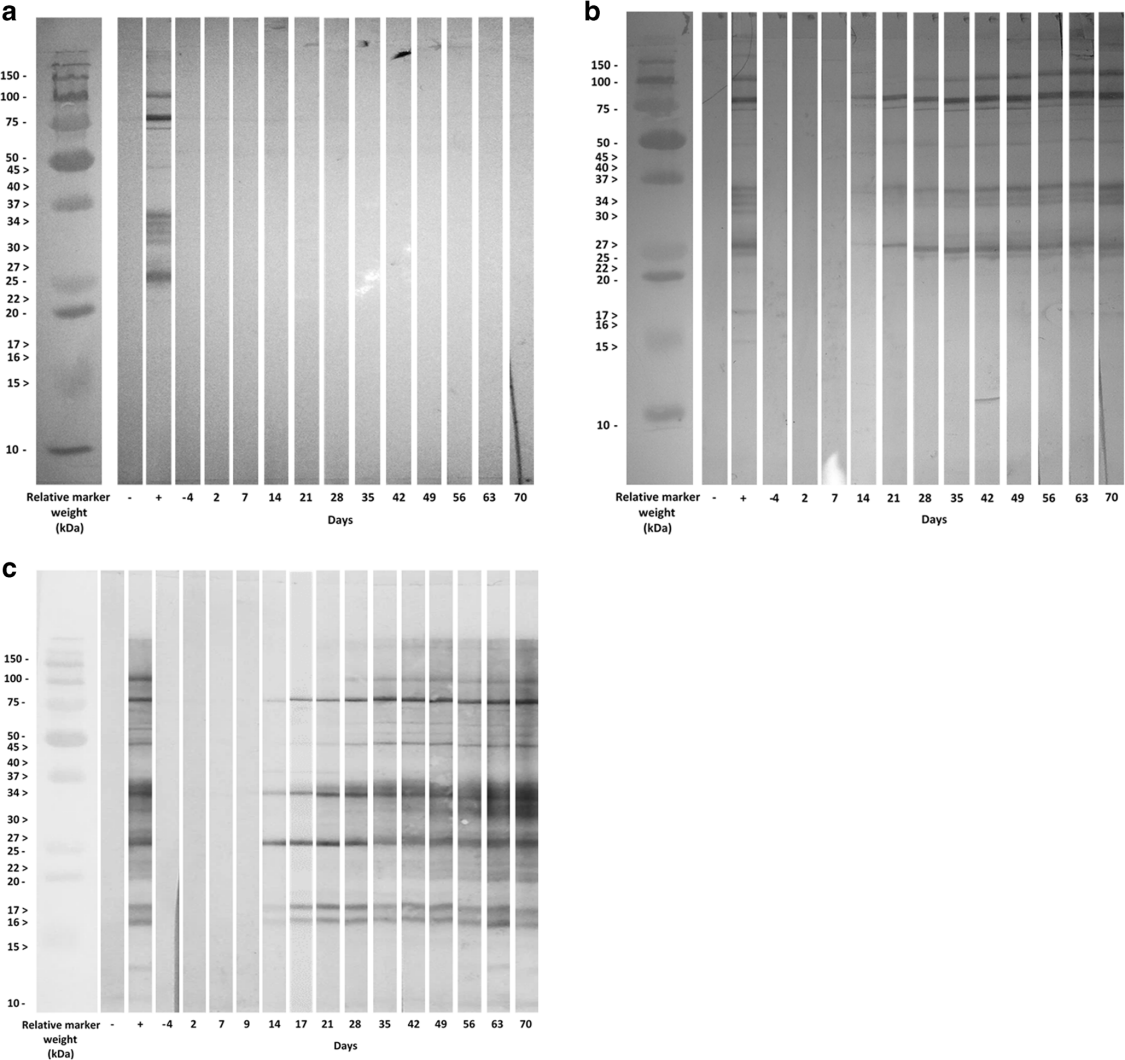
Kinetics of the immunoblot reaction against *B. besnoiti* tachyzoite antigens in each rabbit group. The immunoblot pattern is presented for one rabbit per group. Rabbits were experimentally subcutaneously challenged with 66 μg of ovalbumin (group C, **a**), 6.10^6^ tachyzoites (group T, **b**) and 6.10^6^ bradyzoites (group B, **c**) of *B. besnoiti* at day 0. Control sera (−) were obtained from rabbits inoculated subcutaneously with 66 μg of ovalbumin. Positive sera (+) were obtained from one rabbit inoculated subcutaneously with 6.10^6^ bradyzoites of *B. besnoiti*. Ten tachyzoite antigens recognized by *B. besnoiti* naturally infected cows are indicated by arrowheads

**Fig. 3:**
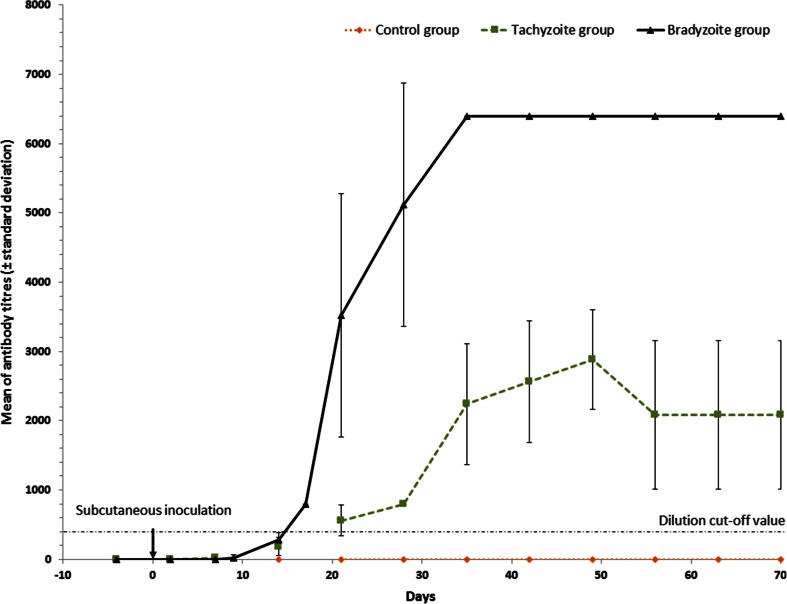
Kinetics of the mean humoral immune response (± *standard deviation bars*) from each rabbit group. Rabbits were experimentally subcutaneously challenged with 66 μg of ovalbumin (group control), 6.10^6^ tachyzoites (group tachyzoites) and 6.10^6^ bradyzoites (group bradyzoites) of *B. besnoiti* at day 0. The *dashed lines* indicate the cut-off value for IgG-IFAT (200). Two dates (day 9 and day 17) were missing for control and tachyzoite groups

No positive seroconversion occurred in group C as determined by WB (Table 1, Fig. 2a) and by IFAT (titre <100, Fig. 3).

**Table 1:** The number of seropositive conversion in each group of rabbits from pre-challenge (day –4) to euthanasia (day 70) determined by Western blot with non-reduced *B. besnoiti* tachyzoite antigens

	Day													
Group	–4	2	7	9	14	17	21	28	35	42	49	56	63	70
Control (*n* = 6)	0/6	0/6	0/6	*n.d.*	0/6	*n.d.*	0/6	0/6	0/6	0/6	0/6	0/6	0/6	0/6
Tachyzoites (*n* = 5)	0/5	0/5	0/5	*n.d.*	4/5	*n.d.*	5/5	5/5	5/5	5/5	5/5	5/5	5/5	5/5
Bradyzoites (*n* = 5)	0/5	0/5	0/5	0/5	5/5	5/5	5/5	5/5	5/5	5/5	5/5	5/5	5/5	5/5

Rabbits were experimentally subcutaneously inoculated at day 0 with 66 μg of ovalbumin (control group), 6.10^6^ tachyzoites (tachyzoite group) and 6.10^6^ bradyzoites (bradyzoite group)

*n.d*. not done

One rabbit out of five in group T remained negative by immunoblot at 14 dpi (Table 1, Fig. 2b); meanwhile, all rabbits inoculated with bradyzoites were seropositive at this date with low antigenic bands below 20 kDa recognition (Table 1, Fig. 2c). All positive seroconversions occurred at day 21 post-inoculation in group T (Table 1). From this date, all rabbits of groups T and B remained seropositive by WB until the end of the experiment (Table 1). The WB-positive pattern was different between groups B and T. All rabbits belonging to group B exhibited bands of low molecular weight (below 20 kDa) at 14 dpi while only two rabbits in group T showed these antigenic bands at 21 and 28 dpi.

The kinetics of the antibody response following the injections of tachyzoites or bradyzoites is demonstrated in Fig. 3. Two rabbits from group T showed seroconversion at day 14 after the inoculation by tachyzoites with antibody titres of 200 and 400. All rabbits from group T had seroconversion equal or greater than the IFAT cut-off value at 21 dpi, achieving the highest peak around the 49th day with antibody titres ranging from 1600 to 3200 (Fig. 3). Then, the mean antibody titre decreased to a mean titre of 2080 ranging from 800 to 3200 and persisted until 70 dpi (Fig. 3). The remaining five rabbits of group B showed positive seroconversion at 17 dpi, earlier than group T, with antibody titres of 200 for two rabbits and 400 for three other rabbits (Fig. 3). The rise of antibody levels was continued until 35 dpi. Then, the antibody titre remained at the highest dilution (6400) in group B and was higher than the mean of the antibody level of group T until euthanasia at 70 dpi. However, the difference was not significant after Bonferroni correction (Fig. 3).

### qPCR examinations of blood

No *B. besnoiti* DNA was evidenced in group C throughout the study. Detection of parasite DNA occurred only twice in group T on two different rabbits (T02 and T03) at 2 dpi and 21 dpi with moderate Ct values (Table 2). One rabbit (B01) showed positive result 7 dpi with high Ct value of 38. All rabbits from group B exhibited positive Ct values at 9 and 14 dpi with values ranging from 32 to 38.9 (Table 2). The mean Ct values between these two dates (35.9 ± 1.7 at 9 dpi and 36 ± 2.9 at 14 dpi) were not significantly different (*p* = 1). The detection of parasite DNA at 9 dpi was concomitant to the rise of rectal temperature in this group. The rectal temperature returned to normal values; whereas, parasite DNA was again evidenced in the blood of rabbits of group B at 14 dpi (all rabbits), 17 dpi for two rabbits and 21 dpi for one rabbit (Table 2). Regarding the two latest dates, Ct values were high and higher than 37 (Table 2). After this date, no *B. besnoiti* DNA was detected in the blood until the end of the experiment.

**Table 2:** Kinetics of *B. besnoiti* DNA determined by quantitative PCR in the blood of inoculated rabbits from pre-challenge to euthanasia

	Tachyzoite-infected rabbits					Bradyzoite-infected rabbits				
Date	T01	T02	T03	T04	T06	B01	B02	B04	B05	B06
–4	–	–	–	–	–	–	–	–	–	–
2	–	35.4	–	–	–	–	–	–	–	–
7	–	–	–	–	–	38	–	–	–	–
9	n.d.	n.d.	n.d.	n.d.	n.d.	34.1	36	34.4	37.8	37.6
14	–	–	–	–	–	35.8	32	34.7	38.6	38.9
17	n.d.	n.d.	n.d.	n.d.	n.d.	–	–	37.9	37.5	–
21	–	–	35	–	–	–	37.1	–	–	–
28	–	–	–	–	–	–	–	–	–	–
35	–	–	–	–	–	–	–	–	–	–
42	–	–	–	–	–	–	–	–	–	–
49	–	–	–	–	–	–	–	–	–	–
56	–	–	–	–	–	–	–	–	–	–
63	–	–	–	–	–	–	–	–	–	–
70	–	–	–	–	–	–	–	–	–	–

Ct values are reported in the table. Rabbits were experimentally subcutaneously infected with either 6.10^6^ *B. besnoiti* tachyzoites (tachyzoite group) or bradyzoites (bradyzoite group) at day 0

*n.d*. not done

–: no Ct

### qPCR examination on the skin and tissues

No qPCR examinations were positive for *B. besnoiti* genomic DNA in group C and in group T (Tables 3 and 4). Thirty-seven DNA amplifications of *B. besnoiti* out of 120 were positive in group B. At least one sample was qPCR positive in each rabbit of group B (Tables 3 and 4). Skin and tissue samples showed 15 positive results out of 50 and 22 positive results out of 70, respectively. The Ct values ranged from 24.6 to 38 with a median Ct value of 30.9 (Tables 3 and 4). The mean Ct value was 30.3 ± 1.8 for the skin samples and was significantly lower than the mean Ct value (33.6 ± 4.2) for the tissue sampling (*p* = 0.008). The rabbit B06 was PCR negative for all skin samples. No detection of parasite DNA occurred in the backline, umbilicus and right shoulder for all group B rabbits (Table 3). The lowest Ct value (26.8) for the skin samples was found in the hind leg of the B05 and the highest (38) in the teat of B01. Parasite DNA was recovered at this location for four rabbits out of five. Other qPCR-positive skin samples were the fore leg, right thigh, neck, teat right eyelid and right flank with Ct values from 28.6 to 38 (Table 3). The two females (B02 and B04) exhibited the same pattern of parasite DNA distribution in skin samples (Table 3). The same results were observed for males B01 and B05 regarding skin samples. An additional positive qPCR result was found at the right flank for the B01 (Table 3). For the tissue samples, the nasal mucosa was the only positive tissue in all rabbits and displayed high levels of parasite DNA with Ct values of 24.6 and 25.2 for rabbits B01 and B02 (Table 4). The nasal mucosa, tracheal mucosa and lung samples recovered altogether 12 positive qPCR amplifications out of 22. Positive results varied from one location to another and one rabbit to another without clear pattern of distribution (Table 4). Other tissues that yielded positive qPCR results were the heart, pancreas, right eye and reproductive system with ovary or testis, vagina or penis and scrotum or vulva (Table 4). No parasite DNA was found in the liver, gallbladder, kidney, spleen and diaphragm.

**Table 3:** Quantitative PCR Ct values of *B. besnoiti* in skin samples of inoculated rabbits at euthanasia

	Tachyzoite-infected rabbits					Bradyzoite-infected rabbits				
Skin location	T01	T02	T01	T02	T01	B01	B02	B04	B05	B06
Right fore leg	–	–	–	–	–	31.1	–	–	28.6	–
Right hind leg	–	–	–	–	–	29.9	30.9	29.8	26.8	–
Right thigh	–	–	–	–	–	–	30.1	33.8	–	–
Backline	–	–	–	–	–	–	–	–	–	–
Right flank	–	–	–	–	–	28.7	–	–	–	–
Umbilicus	–	–	–	–	–	–	–	–	–	–
Neck	–	–	–	–	–	–	32.5	30.3	–	–
Right shoulder	–	–	–	–	–	–	–	–	–	–
Right eyelid	–	–	–	–	–	31	–	–	28.2	–
Teat	–	–	–	–	–	38	–	–	31.9	–

Ct values are reported in the table at day 70. Rabbits were experimentally subcutaneously infected with either 6.10^6^ *B. besnoiti* tachyzoites (tachyzoite group) or bradyzoites (bradyzoite group) at day 0

–: no Ct

**Table 4:** Quantitative PCR Ct values of *B. besnoiti* in tissues of inoculated rabbits at euthanasia

	Tachyzoite-infected rabbits					Bradyzoite-infected rabbits				
Tissue location	T01	T02	T01	T02	T01	B01	B02	B04	B05	B06
Nasal mucosa	–	–	–	–	–	24.6	25.5	31.2	29.2	35.9
Tracheal mucosa	–	–	–	–	–	–	37	34.6	37.7	–
Lung	–	–	–	–	–	32.2	36.7	35.2	35.9	–
Heart	–	–	–	–	–	35.6	–	–	–	–
Pancreas	–	–	–	–	–	38	–	–	–	–
Liver	–	–	–	–	–	–	–	–	–	–
Gallbladder	–	–	–	–	–	–	–	–	–	–
Kidney	–	–	–	–	–	–	–	–	–	–
Spleen	–	–	–	–	–	–	–	–	–	–
Diaphragm	–	–	–	–	–	–	–	–	–	–
Right eye	–	–	–	–	–	32	36.7	–	35.2	–
Ovary or testis	–	–	–	–	–	–	35 (ovary)	–	38 (testis)	–
Vagina or penis	–	–	–	–	–	27.9 (penis)	–	–	27.6 (penis)	–
Scrotum/vulvar mucosa	–	–	–	–	–	–	–	–	38	–

Ct values are reported in the table at day 70. Rabbits were experimentally subcutaneously infected with either 6.10^6^ *B. besnoiti* tachyzoites (tachyzoite group) or bradyzoites (bradyzoite group) at day 0

–: no Ct

The same PCR examinations were performed on both dead rabbits in group T (T05) and group B (B03). Parasite DNA was recovered in the right flank of the T05 rabbit which was the site of parasite injection of the parasite with a Ct value of 33.1. One *B. besnoiti* DNA amplification (Ct value of 35.5) was observed for the B03 rabbit in the nasal mucosa at 1 dpi.

### Histopathology and immunohistochemistry

**Fig. 4:**
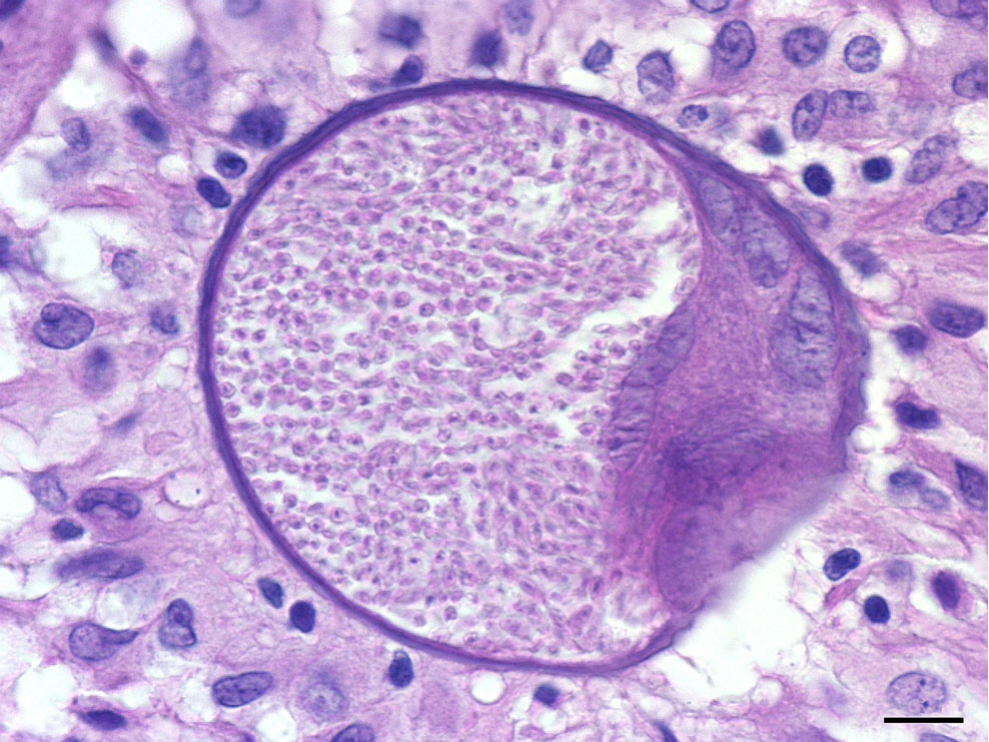
Histopathological identification of *B. besnoiti* cyst (haematoxylin and eosin stain, ×1000). The sample was taken at 70 dpi from the fore leg dermis of the B05 rabbit. Tissue cyst of *B. besnoiti* in collagenic dermis was spherical in shape, thick walled and contained a single maximally developed parasitophorous vacuole with thousands of bradyzoites. Note the several host cell nuclei and nucleoli. A granulomatous inflammatory infiltrate (predominantly macrophages and lymphocytes) surrounded the cyst. *Bar* = 10 μm

**Fig. 5:**
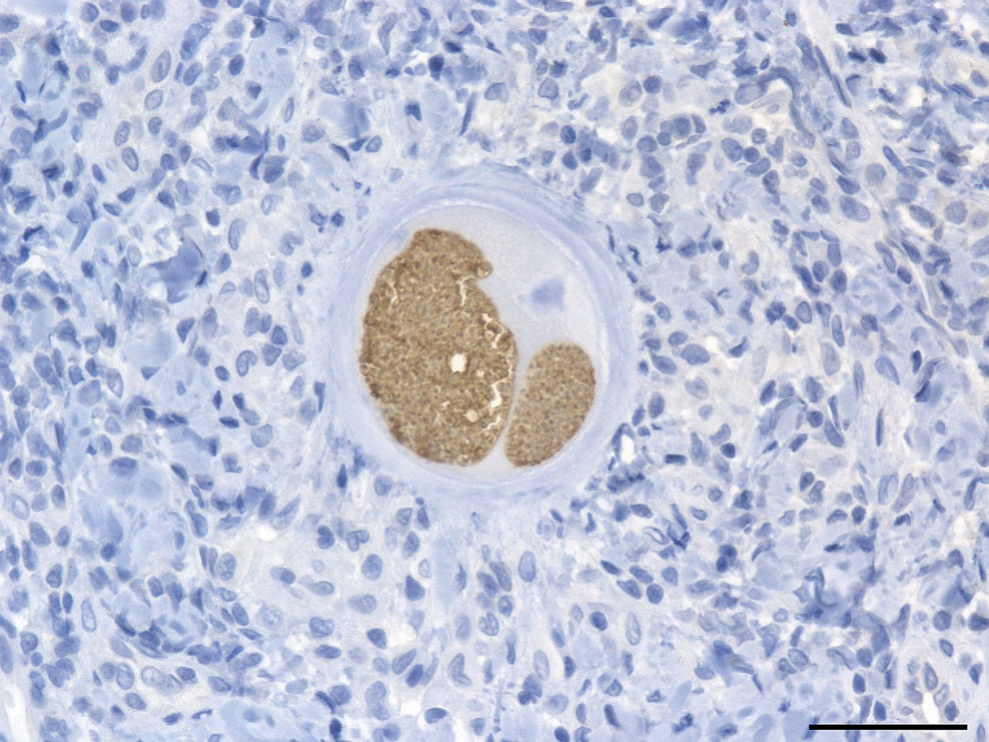
Anti-*B. besnoiti* immunohistochemistry with Harris haematoxylin counterstain of cyst (×400). The cyst was observed at 70 dpi in nasal mucosa of the B05 rabbit. *B. besnoiti* cyst displayed a strong specific brown labelling of bradyzoites in two parasitophorous vacuoles developed in the enlarged host cell. The cyst wall as well as the host cell and nucleus were completely negative at immunohistochemistry. *Bar* = 50 μm

Cysts were recovered in the 11 samples exhibiting Ct values below 30 (tables 3 and 4) in group B and corresponding to the fore and hind leg, the right flank, the nasal mucosa, the right eyelid and the penis (Fig. 4). They were readily identified as *B. besnoiti* cysts with anti-*B. besnoiti* immunohistochemistry (Fig. 5).

## Discussion

The first aim of this study was to reproduce the clinical course of bovine besnoitiosis in rabbit by subcutaneous injection of *B. besnoiti*. Several previous attempts on rabbit inoculations have been undertaken, but results have showed discrepancies in clinical signs and lesions. The first inoculations of rabbits by *B. besnoiti* tachyzoites were performed by Pols (1954a) in South Africa. One rabbit was inoculated by intraperitoneal injection of 5 ml of ox blood during the acute stage, and, the same day, a second rabbit was infected by subcutaneous injection of 10 ml of blood from the same ox donor. Then, serial passages on rabbits were achieved by either intravenous, intraperitoneal or subcutaneous blood injection of different volumes (from 2 to 10 ml). The inoculum sizes of tachyzoites were unquantified. The first clinical sign to be reported was fever starting 3 to 16 days after the infection (up to 41.7 °C) and lasting 2 to 4 days before death of all rabbits. Oedemas of the hind limbs and the base of the ear were also reported. Subsequent studies based on inoculations to rabbits of tachyzoites or bradyzoites by different routes, sources and doses of parasites presented a wide range of clinical signs from absence (Cortes et al. 2006a) to acute symptoms (Bigalke 1960, 1967, 1968; Basson et al. 1970; Neuman and Nobel 1981). The main and recurrent clinical signs during the onset of the fever stage were oedemas (localizations as previously described by Pols (1954a) but also localizations including the vulva, testes, scrotum, penis, ears and faces), necrosis (scrotum and testes) and a fatal outcome with a rate ranging from 20 to 100 % (Bigalke 1960, 1967, 1968; Basson et al. 1970; Neuman and Nobel 1981). Two studies detailed mild and transient symptoms following inoculation of rabbits (Shkap et al. 1987; Basso et al. 2011). In the first experiment, 1.10^7^ tachyzoites cultivated on Vero cells were administered intraperitoneally (Shkap et al. 1987). In the second experiment, two batches of rabbits were subcutaneously inoculated with either 5.10^5^ tachyzoites cultivated on MARC-145 cells or 5.10^5^ bradyzoites recently isolated from a cow (Basso et al. 2011). The symptoms included fever (higher than 40 °C), conjunctivitis and oedematous swelling of the testes and reversed after 20 dpi to the fullest without mortality (Shkap et al. 1987; Basso et al. 2011). Except fever, the clinical signs involved only few animals among inoculated ones. In the present investigation, the rabbits of group B received 6.10^6^ bradyzoites subcutaneously as intermediate dose between those performed by Shkap et al. (1987) and Basso et al. (2011). They exhibited few clinical signs except for fever (up to 40.4 °C) lasting 4 days, transient photophobia and, for one rabbit, catarrh. Two rabbits died 1 to 3 days after inoculation without clinical signs. However, their death could not be attributed undoubtedly to the parasite injection despite the extensive necropsy examination. The toxicity of the inoculum may be responsible for the mortality within 24 h (Bigalke 1967).

Pols (1954a, 1960), Bigalke (1968) and Basson et al. (1970) found tachyzoites in blood smears during the fever stage of the disease contrary to Shkap et al. (1987). Although no direct examination of stained blood smears was achieved in this work, parasite DNA was clearly recovered during the acute stage at 9 and 14 dpi for all rabbits of group B. These results are congruent with those obtained by Basso et al. (2011) at 9 dpi. One rabbit of group T was also qPCR positive at 7 dpi and another one at 21 dpi. This extended presence of parasite in blood was also reported by Pols (1960) until 24 dpi, and, according to the same author, it seems longer than for cattle.

In this study, the presence of parasite DNA was investigated in 25 different locations in the skin and tissues after euthanasia at 70 dpi. Positive results were evidenced for group B in various skin and tissue samples. The classical dermotropism of *B. besnoiti* was recovered in this study (Bigalke 1960, 1967, 1968; Basson et al. 1970). The respiratory system (nasal mucosa, tracheal mucosa and lung) was a frequent site of positive qPCR results. The nasal mucosa was the only tissue exhibiting positive qPCR results for all rabbits of group B with three low Ct values below 30. The third tropism observed involved the genital tract (ovary or testis, vagina or penis and scrotum or vulva mucosa for 3 rabbits out of 5). Other positive tissues provided the sclera of the right eye (three rabbits), the heart (one rabbit) and the pancreas (one rabbit with Ct value of 38). Interestingly, most locations were also reported in naturally infected cattle to be the upper respiratory tract, female distal genital tract and skin (Frey et al. 2013; Liénard et al. 2013b). Positive qPCR results were also found in common voles *Microtus arvalis* experimentally inoculated with bradyzoites in the skin, lung and heart but also in muscle and kidney (Basso et al. 2011). Overall parasite loads were low according to the high Ct values. This result may be explained firstly by the difficulty of *B. besnoiti* establishment and adaptation to heterologous host. Indeed, bradyzoite cysts were scarce in rabbit as reported by previous studies (Bigalke 1960, 1967, 1968; Basson et al. 1970). Secondly, only 50 mg of tissues or skin per localization were used for molecular analyses. The patchy distribution of rare parasite cysts in tissues could lead to failure of parasite DNA amplification despite the sensitivity of qPCR (Schares et al. 2011). Increasing the quantity of tested tissue or skin to 2 g or more followed by pepsin-HCl digestion before DNA extraction may overcome this issue (Basso et al. 2011). Thirdly, the delay between infection and necropsy (i.e. 70 days) may be too short to allow fully mature development of cysts.

The detection of *B. besnoiti* DNA does not mean that the parasites are viable and infectious for a novel host. According to Pols (1954b), trophozoites can be detected at 16 to 18 days after the inoculation into a formed cytoplasmic vacuole of 8 μm of the host cell (mainly histiocytes). Cysts enlarge and then measure from 50 to 200 μm at 9 weeks with an outer hyaline capsules. More than one trophozoite can invade a cell, so they develop in the same cell cytoplasm, enclosed in its own membrane (Pols 1954b). Skin and tissue cysts marked by specific immunohistochemistry were in qPCR-positive samples with Ct <30 exhibiting similar morphology as described by Pols (1954b). They provided also additional proof of the installation and viability of *B. besnoiti* in rabbit tissues and skin. Moreover, to date and since Basso et al. (2011), no bradyzoite cysts have been recorded in various immunocompetent species of rodents and lagomorphs following experimental infection (Neuman and Nobel 1981; Shkap et al. 1987; Cortes et al. 2006b; Basso et al. 2011).

The second aim of this study was to compare the pathogenicity of bradyzoites and those of high passage-level tachyzoites on Vero cells in rabbits. The most striking finding of this study was the apparent safety of the tachyzoite stage to rabbits, at least after a long period of in vitro culture. Another aspect supporting this view was the difference of serological response observed between the two groups of inoculated rabbits. Meanwhile, all rabbits in the tachyzoite group remained asymptomatic and showed no cysts or positive qPCR results; they developed antibodies against *B. besnoiti*. However, the seroconversion was more precocious in the bradyzoite group, at least 2 weeks after the inoculation as shown by Western blot and IFAT. The features of the serological responses were also more pronounced in the bradyzoite group. Immunoblotting analysis exhibited earlier (at 14 dpi) and higher number of antigenic bands of low weight (≤20 kDa) recognitions in this group in comparison to the tachyzoite group. In the same way, IFAT showed earlier and higher peak (≥6400 at 35 dpi) and more stable antibody titres until euthanasia (≥6400 at 70 dpi). In the tachyzoite group, the peak of the antibody level was lower (2880) and occurred at 49 dpi before decreasing to 2080 and remaining stable till the end of the experiment. The evolution of the antibody titre was slightly different in the study of Basso et al. (2011). All rabbits were seropositive at 3 weeks post-infection (wpi) and the mean antibody level was higher in the tachyzoite group until 5 wpi. After this date, and until 18 wpi, the antibody titre was higher in the bradyzoite group. No difference in the Western blot patterns was reported by Basso et al. (2011) for tested rabbits of both groups contrary to our results with more frequent and precocious low antigenic bands in the group B. The serological response was also more pronounced in bradyzoite-inoculated mice (Basso et al. 2011). All four mice have seroconverted with titre peak from 200 to 1600, but only two out of four tachyzoite-inoculated mice were positive with low positive antibody level ≤200 (Basso et al. 2011). Congruent with our results, a better stimulation of the host immune system seems to occur with bradyzoites. However, a major difference was the absence of detected cysts by Basso et al. (2011). In cattle, it was suggested that bradyzoite cysts could boost the immune response by longer exposure to *B. besnoiti* and by possible re-exposure following relapse of parasite by disruption of cyst wall (Basson et al. 1970; Fernández-García et al. 2009; Liénard et al. 2011; Schares et al. 2013; Gollnick et al. 2015).

In our study, animals were inoculated with the same dose and by the same route suggesting a pathogenicity attenuation of the tachyzoite stage without cyst development. The rapid loss of cyst-forming ability was also known for *Besnoitia jellisoni* and occurred after 20 acute passages in mice only (Frenkel et al. 1976). Moreover, virulence attenuations are also reported for closest relative *B. besnoiti* species such as *T. gondii* and *N. caninum* according to the host cell line, the parasite strain and the number of in vitro passages (Khordadmehr et al. 2013). The attenuation of *B. besnoiti* tachyzoite virulence specifically addressed to the effect of Vero cell cultivation needs more investigations. The comparisons on rabbits with the same isolate at the bradyzoite stage and at the tachyzoite stage but at different numbers of passages on Vero cells could be a way of demonstration of the effect of long-lasting in vitro cell culture.

The strain notion remains badly examined for cattle *B. besnoiti* and could explain the diversity of virulence in various tested hosts and tolerance to in vitro culture. To support this opinion, it is noticeable but yet unexplained that some isolates have failed to grow on Vero cells; meanwhile, others have succeeded whatever their source or the used stage (Schares et al. 2009).

To conclude, our results confirm that rabbits are susceptible to subcutaneous inoculation of *B. besnoiti* bradyzoites with mild clinical signs and formation of cysts. Although some warnings must be taken with a heterologous model, its application could be promising for immunological studies, tests of new treatments or vaccines for example. On the other hand, evidence has been collected suggesting the safety of *B. besnoiti* tachyzoites after 125 passages on Vero cells from an isolate originating of an enzootic area of cattle besnoitiosis. Further studies are required to confirm the vaccine ability of our isolate perpetuated on Vero cells.
